# Analysis of variations in the bronchovascular pattern of the lingular segment to explore the correlations between the lingular segment artery and left superior division veins

**DOI:** 10.3389/fsurg.2023.1173602

**Published:** 2023-04-20

**Authors:** Zhikai Li, Wenbo Wu, Yuhong Kong, Shuangqing Chen, Xiaopeng Zhang

**Affiliations:** ^1^ Graduate School, Hebei Medical University, Shijiazhuang, China; ^2^ Department of Thoracic Surgery, Hebei General Hospital, Shijiazhuang, China; ^3^ Graduate School, Hebei North University, Zhangjiakou, China

**Keywords:** lingular segment (LS), anatomical variation, lingular segment artery (LSA), left superior division veins (LSDV), non-small cell lung carcinoma (NSCLC), segmentectomy, video-assisted thoracoscopic surgery (VATS)

## Abstract

**Background:**

With the development of anatomical segmentectomy, the thoracic surgeons must master the anatomical variations of the pulmonary bronchi and vessels. However, there are only a few reports showing anatomic variations of the lingular segment (LS) using three-dimensional computed tomography bronchography and angiography (3D-CTBA). Thus, the present study aimed to analyze the bronchovascular patterns of the LS and explore the correlation between the lingular segment artery (LSA) and left superior division veins (LSDV).

**Materials and methods:**

The 3D-CTBA data of the left upper lobe (LUL) were collected from patients who had undergone lobectomy or segmentectomy at Hebei General Hospital between October 2020 and October 2022. We reviewed the clinical characteristics and variations in bronchi and pulmonary vessels and grouped them according to different classifications.

**Results:**

Among all 540 cases of 3D-CTBA, the branching patterns of LSA included 369 (68.3%) cases with the interlobar origin, 126 (23.3%) cases with the interlobar and mediastinal origin, and 45 (8.3%) cases with the mediastinal origin. The branching pattern of LSDV could be classified into three forms: Semi-central vein type (345/540, 63.9%), Non-central vein type (76/540, 14.1%), and Central vein type (119/540, 22.0%). There were 51 cases (9.4%) with Non-central vein type, 50 cases (9.3%) with Central vein type, 268 cases (49.6%) with Semi-central vein type in the interlobar type, and 7 cases (1.3%) with Non-central vein type, 9 cases (1.7%) with Central vein type, 29 cases (5.4%) with Semi-central vein type in the mediastinal type. Moreover, the Non-central vein type, the Central vein type, and the Semi-central vein type accounted for 18 (3.3%), 60 (11.1%), and 48 (8.9%) in the interlobar and mediastinal type. Combinations of the branching patterns of the LSA and LSDV were significantly dependent (*p* < 0.005). The combinations of the interlobar and mediastinal type with the Central vein type, and the interlobar type and the mediastinal type with the Semi-central vein type were frequently observed.

**Conclusions:**

This study found the relationship between the LSA and LSDV and clarified the bifurcation patterns of the bronchovascular in the LS. Our data can be used by thoracic surgeons to perform safe and precise LS segmentectomy.

## Introduction

High-resolution computed tomography (HRCT) technology is widespread nowadays, resulting in increased detection of ground-glass opacities (GGOs). The JCOG0804 study showed that sublobar resection with enough surgical margin was feasible and effective for GGO-dominant peripheral lung cancer (1). Therefore, video-assisted thoracoscopic surgery (VATS) sublobar resection should be the first choice of mode of operation for treating early-stage non-small cell lung carcinoma (NSCLC) (1–5). The main forms of sublobar resection currently include wedge resection and anatomical segmentectomy. However, VATS segmentectomy is more difficult than a standard lobectomy because of the anatomical diversity of the bronchovascular pattern of the lung segment. Thus, thoracic surgeons must understand anatomical variations in the pulmonary bronchi and vessels.

It is challenging to distinguish anatomical variations of pulmonary bronchi and vessels from conventional two-dimensional (2D) CT images. Fortunately, three-dimensional (3D) preoperative reconstruction from 2D images allows thoracic surgeons to identify pulmonary anatomical structures before the procedure. Some studies revealed that an intraoperative lingular segment artery (LSA) injury was more common during the left upper lobe (LUL) lobectomy (6, 7). However, there are only a few studies describing anatomic variations of the lingular segment (LS) using three-dimensional computed tomography bronchography and angiography (3D-CTBA) (8). In our clinical practice, the anatomical variation of the lingular segment bronchus (LSB) does not accordant with the previous reports (9, 10). Moreover, the more detailed classification and occurrence probability of the branching patterns of LSA has been rarely involved in previous studies (11–16). The present study aimed to analyze the bronchovascular pattern of the LS by using data derived from 3D-CTBA and provided a new perspective to understand pulmonary anatomical variations in LS. Furthermore, we analyzed the classification of the left superior division veins (LSDV) to explore the associated pulmonary anatomical characteristics of LSA.

## Methods

### Patient preparation and reconstruction of 3D-CTBA

Between October 2020 and October 2022, 540 patients (248 men and 292 women) who were scheduled to undergo surgeries for treating lesions in LUL were enrolled in this study. The mean age was 56 years. These patients underwent routine chest-enhanced CT examinations preoperatively at Hebei General Hospital. The CT data were imported into the reconstruction software (InferOperate Thorax Planning), which converted the data into 3D virtual models of the lungs, including segments, subsegments, lesions, bronchi, and vessels. Error identification and defects in distal bronchial imaging were manually modified. Variations in the LSA, LSB, lingular division veins (LLDV) and LSDV were analyzed and summarized. All procedures involving human participants in this study were in accordance with the Declaration of Helsinki (revised in 2013). This retrospective study was approved by the Research Ethics Committee at Hebei General Hospital (no. 2022119). The need for patient consent was waived because of the retrospective nature of the study.

### Definition of the LSB

**Figure 1:**
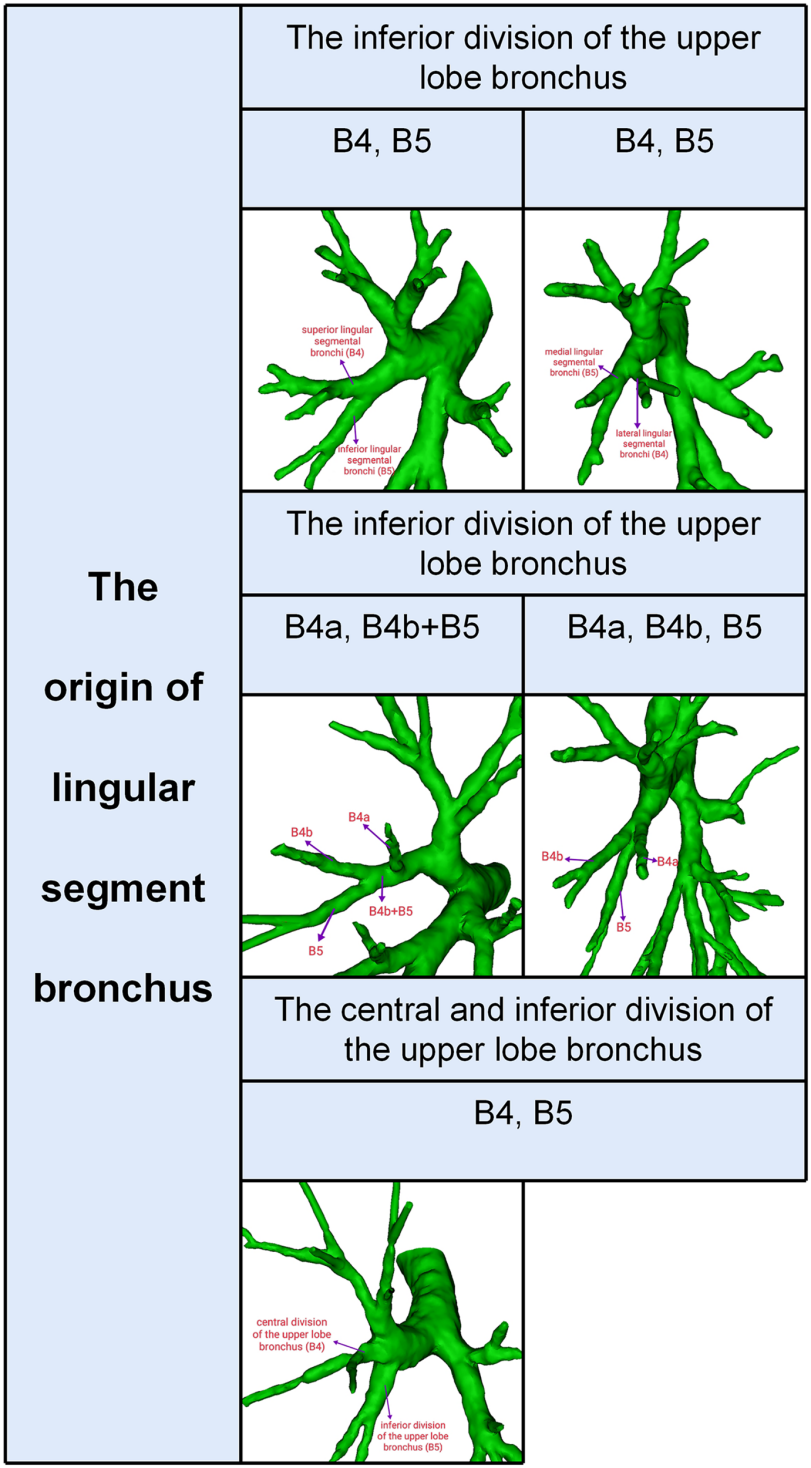
3D reconstruction model of branching patterns of the lingular segment bronchus.

Segmental and subsegmental bronchi of LS were usually named (Figure 1): B4 is the superior lingular segmental bronchus that divides into lateral (B4a) and medial ramus (B4b); B5 is the inferior lingular segmental bronchus that is further divided into superior (B5a) and inferior ramus (B5b). However, the lingular segment bronchi bifurcate into lateral lingular (B4) and medial lingular (B5) segmental bronchus in rare conditions (Figure 1).

### Definition of the LSDV type

**Figure 2:**
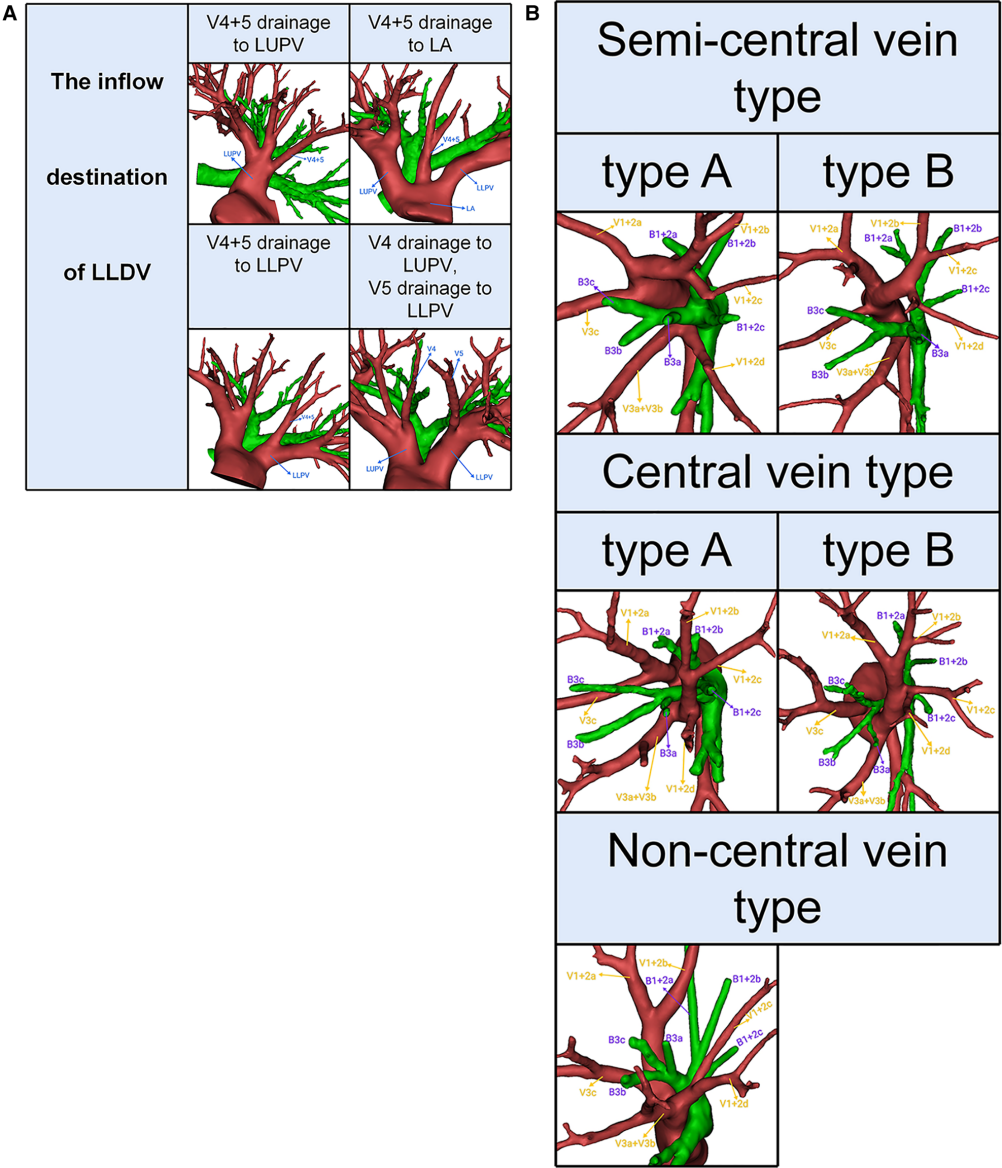
(**A**) 3D reconstruction model of branching patterns of the left lingular division veins. (**B**) 3D reconstruction model of branching patterns of the left superior division veins.

The left upper pulmonary veins (LUPV) contain LSDV and LLDV. The LSDV bifurcates into apicoposterior segmental vein (V1 + 2) and anterior segmental vein (V3). The LLDV bifurcates into superior lingular segmental vein (V4) and inferior lingular segmental vein (V5) (Figure 2A). V1 + 2 includes four branches as follows: V1 + 2a (between S1 + 2a and S3c), V1 + 2b (between S1 + 2a and S1 + 2b), V1 + 2c (between S1 + 2b and S1 + 2c) and V1 + 2d (between S1 + 2c and S3a). Furthermore, with the exception of V3b, V3 is not anatomically involved in the pulmonary segmental structure. Thus, to avoid confusion, the V3 veins were excluded from the classification of LSDV (17). The patterns of LSDV are divided into three types: Semi-central vein type, Non-central vein type and Central vein type (Figure 2B). Semi-central vein type can be further classified into two anatomical categories: type A, V1 + 2a, V1 + 2b, and V1 + 2c shares common trunk and drains into LUPV above B3 on the mediastinal side; type B, V1 + 2a, V1 + 2b, V1 + 2c, and V1 + 2d shares a common trunk and drains into LUPV above B3 on the mediastinal side (Figure 2B). Similarly, the Central vein type was further subclassified into two subtypes: type A, V1 + 2b, V1 + 2c, and V1 + 2d shares a common trunk and drains into LUPV below B3; type B, V1 + 2a, V1 + 2b, V1 + 2c, and V1 + 2d shares a common trunk and drains into LUPV below B3 (Figure 2B). For the Non-central vein type, V1 + 2a and V1 + 2b drain into the LUPV above B3 on the mediastinal side, and V1 + 2c and V1 + 2d drain into LUPV below B3 (Figure 2B).

### Definition of the LSA type

**Figure 3:**
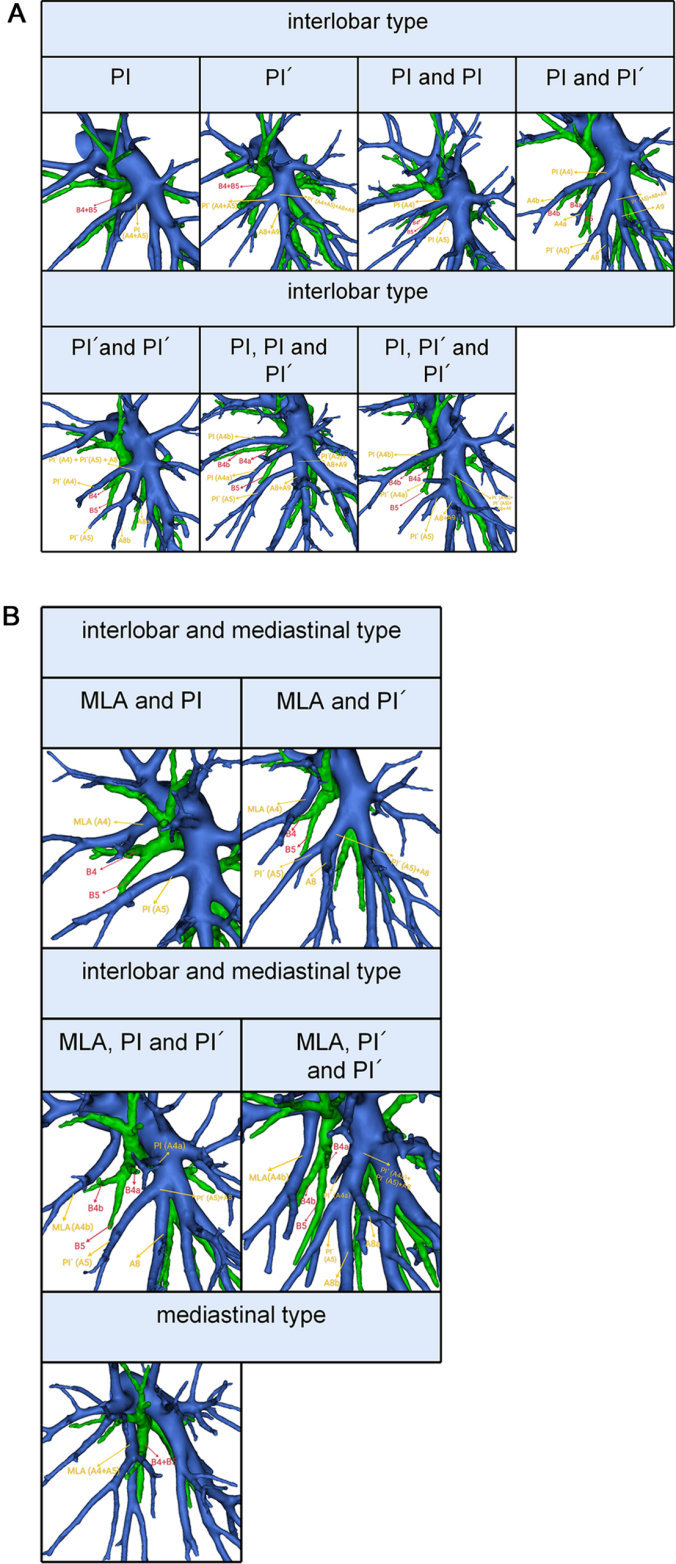
(**A**) 3D reconstruction model of branching patterns of the lingular segment artery in the interlobar type. (**B**) 3D reconstruction model of branching patterns of the lingular segment artery in the mediastinal type and the interlobar and mediastinal type.

According to the origin of LSA, the branching patterns of LSA were categorized into three types as follows: interlobar type, in which all arteries supplying the LS originate from pars interlobaris; mediastinal type, in which all arteries supplying the LS originate from pars mediastinalis; and interlobar and mediastinal type, in which arteries supplying the LS originate from pars interlobaris and pars mediastinalis (Figure 3). Thus, the LSA was nominated as mediastinal lingular arteries (MLA) in the mediastinal type (Figures 3A,B). The interlobar portion of the left pulmonary artery was divided into two types (15): the superior portion, which locates above the terminal branches to the lower lobe; the inferior portion, which locates beneath the terminal branches to the lower lobe. We defined the LSA as PI originating from the superior interlobar portion and PÍ from the inferior interlobar portion, that is, from A8 or the common trunk of A8 and A9 (Figures 3A,B).

### Statistics

All statistical analyses were conducted using SPSS 23.0 (SPSS, Chicago, IL, USA). Qualitative data were expressed as the number of cases (percentage). The Pearson Chi-Square test was used to assess the significance of dependencies between the groups. A *p*-value less than 0.05 was considered statistically significant.

## Results

### Branching patterns of LSB

According to the origins of LSB, two types can be defined (Table 1, Figure 1): type 1, in which a common trunk of B4 + B5 comes from the inferior division of the upper lobe bronchus (95.6%); type 2, in which B4 comes from the central division of the upper lobe bronchus and B5 comes from the inferior division of the upper lobe bronchus (4.4%). The type 1 was further divided into three subtypes (Table 1, Figure 1): subtype I (B4, B5), subtype II (B4a, B4b + B5), subtype III (B4a, B4b, B5). These subtypes accounted for 65.0%, 20.4%, and 10.2%, respectively. In the subtype I (Figure 1), the branching of LSB bifurcated into lateral lingular (B4) and medial lingular (B5) segmental bronchus in 12 cases (2.2%), while superior (B4) and inferior lingular (B5) segmental bronchus in 339 cases (62.8%).

**Table 1:** Branching patterns of the lingular segment bronchus.

The origin of lingular segment bronchus	Our study (*n* = 540)		Maki (*n* = 539)		Wang (*n* = 166)		Deng (*n* = 103)	
	NO.	%	NO.	%	NO.	%	NO.	%
The inferior division of the upper lobe bronchus	516	95.6	539	100.0	166	100.0	103	100.0
B4, B5	351	65.0	495	91.8	129	77.7	103	100.0
B4a, B4b + B5	110	20.4	44	8.2	31	18.7	NR	-
B4a, B4b, B5	55	10.2	NR	-	6	3.6	NR	-
The central and inferior division of the upper lobe bronchus	24	4.4	NR	-	NR	-	NR	-
B4, B5	24	4.4	NR	-	NR	-	NR	-

NR, the type was not referred.

### Branching patterns of LLDV

Patients can be divided into one of the following four types based on the inflow destination of LLDV (Table 2, Figure 2A): type A, V4 + 5 drains into LUPV (96.3%); type B, V4 + 5 drains into left atrium (LA) (1.3%); type C, V4 + 5 drains into left lower pulmonary veins (LLPV) (0.4%); type D, V4 drains into LUPV, V5 drains into LLPV (2.0%).

**Table 2:** Branching patterns of the left lingular division veins.

The inflow destination of LLDV	Our study (*n* = 540)		Maki (*n* = 539)	
	NO.	%	NO.	%
V4 + 5 drains into LUPV	520	96.3	513	95.2
V4 + 5 drains into LA	7	1.3	4	0.7
V4 + 5 drains into LLPV	2	0.4	6	1.1
V4 drains into LUPV, V5 drains into LLPV	11	2.0	16	3.0

### Branching patterns of LSDV

Semi-central vein type was found in 345 patients (63.9%); Non-central vein type in 76 patients (14.1%); Central vein type in 119 patients (22.0%) (Table 3, Figure 2B).

**Table 3:** Branching patterns of the left superior division veins.

	Our study (*n* = 540)		Chen (*n* = 404)	
	NO.	%	NO.	%
Semi-central vein type	345	63.9	254	62.9
Non-central vein type	76	14.1	36	8.9
Central vein type	119	22.0	114	28.2

### Branching patterns of LSA

The branching patterns of LSA were sorted into interlobar type (68.3%), mediastinal type (8.3%), and interlobar and mediastinal type (23.3%) (Table 4, Figure 3). According to the location of the LSA branching in the interlobar portion of the left pulmonary artery and the number of the LSA, the interlobar type was further divided into seven subtypes (Table 4, Figure 3A): subtype 1, PI (45.7%); subtype 2, PÍ (2.6%); subtype 3, PI and PI (11.5%); subtype 4, PI and PÍ (7.0%); subtype 5, PÍ and PÍ (0.2%); subtype 6, PI, PI and PÍ (0.4%); subtype 7, PI, PÍ and PÍ (0.9%). Similarly, the interlobar and mediastinal type was classified into four subtypes (Table 4, Figure 3B): subtype 1, MLA and PI (19.2%); subtype 2, MLA and PÍ (2.8%); subtype 3, MLA, PI and PÍ (0.9%); subtype 4, MLA, PÍ and PÍ (0.4%).

**Table 4:** Branching patterns of the lingular segment artery.

	Our study (*n* = 540)		Deng (*n* = 103)		Maki (*n* = 539)		Chen (*n* = 404)	
	NO.	%	NO.	%	NO.	%	NO.	%
Interlobar type	369	68.3	79	76.7	378	70.1	297	73.5
PI	247	45.7	NR	-	NR	-	NR	-
PÍ	14	2.6	NR	-	NR	-	NR	-
PI and PI	62	11.5	NR	-	NR	-	NR	-
PI and PÍ	38	7.0	NR	-	NR	-	NR	-
PÍ and PÍ	1	0.2	NR	-	NR	-	NR	-
PI, PI and PÍ	2	0.4	NR	-	NR	-	NR	-
PI, PÍ and PÍ	5	0.9	NR	-	NR	-	NR	-
Mediastinal type	45	8.3	7	6.8	34	6.3	23	5.7
Interlobar and mediastinal type	126	23.3	17	16.5	127	23.6	84	20.8
MLA and PI	104	19.2	NR	-	NR	-	NR	-
MLA and PÍ	15	2.8	NR	-	NR	-	NR	-
MLA, PI and PÍ	5	0.9	NR	-	NR	-	NR	-
MLA, PÍ and PÍ	2	0.4	NR	-	NR	-	NR	-

NR, the type was not referred.

**Figure 4:**
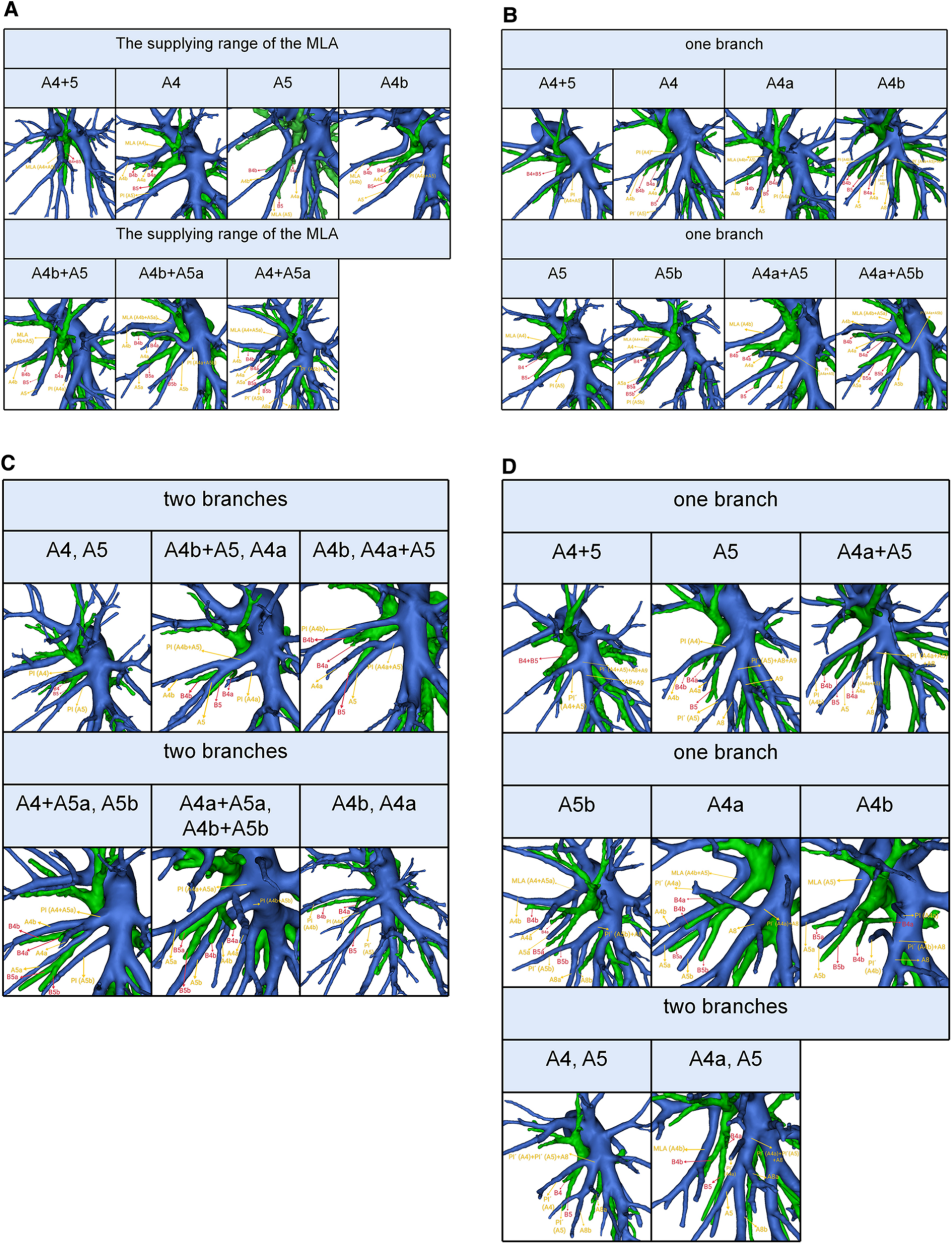
(**A**) 3D reconstruction model of the supplying range of the MLA. (**B**) 3D reconstruction model of the supplying range of the one branch PI. (**C**) 3D reconstruction model of the supplying range of the two branches PI. (**D**) 3D reconstruction model of the supplying range of the PÍ.

The MLA was observed in 171 cases in the present study. Based on the supplying range of the MLA, five types were defined (Table 5, Figure 4A): type A (A4 + A5) was the most common (26.3%); type B (A4) was found in 38 patients (22.2%); type C (A5) was occurred in 7 patients (4.1%); type D (A4b) was observed in 41 patients (24.0%); type E (A4b + A5) was seen in 31 cases (18.1%); type F (A4b + A5a) was the less common (1.8%); type G (A4 + A5a) was present in 6 cases (3.5%).

**Table 5:** The supplying range of the MLA.

	Our study (*n* = 171)	
	NO.	%
A4 + A5	45	26.3
A4	38	22.2
A5	7	4.1
A4b	41	24.0
A4b + A5	31	18.1
A4b + A5a	3	1.8
A4 + A5a	6	3.5

The PI was found in 463 cases. When the composition of LSA contained single branch PI, the supplying range of the PI was classified into eight types (Table 6, Figure 4B): type A, A4 + A5; type B, A4; type C, A4a; type D, A4b; type E, A5; type F, A5b; type G, A4a + A5; type H, A4a + A5b; with frequencies of 53.3% (247/463), 8.2% (38/463), 7.6% (35/463), 2.4% (11/463), 6.5% (30/463), 0.9% (4/463), 7.1% (33/463), and 0.2% (1/463), respectively. When the composition of LSA contained PI of the double branch, types can also be defined according to the supplying range of PI (Table 6, Figure 4C): type 1 (A4, A5); type 2 (A4b + A5, A4a); type 3 (A4b, A4a + A5); type 4 (A4 + A5a, A5b); type 5 (A4a + A5a, A4b + A5b); type 6 (A4b, A4a). These types accounted for 8.0%, 1.3%, 3.0%, 0.9%, 0.2%, and 0.4%, respectively.

**Table 6:** The supplying range of the PI.

	Our study (*n* = 463)	
	NO.	%
One branch		
A4 + A5	247	53.3
A4	38	8.2
A4a	35	7.6
A4b	11	2.4
A5	30	6.5
A5b	4	0.9
A4a + A5	33	7.1
A4a + A5b	1	0.2
Two branches		
A4, A5	37	8.0
A4b + A5, A4a	6	1.3
A4b, A4a + A5	14	3.0
A4 + A5a, A5b	4	0.9
A4a + A5a, A4b + A5b	1	0.2
A4b, A4a	2	0.4

The PÍ was found in 82 cases. When the composition of LSA contained single branch PÍ (Table 7, Figure 4D), the supplying range of the PÍ was sorted into six types: type A (A4 + A5) was found in 14 patients (17.1%); type B (A5) was the most common (53.7%); type C (A4a + A5) was occurred in 10 patients (12.2%); type D (A5b) was observed in 4 patients (4.9%); type E (A4a) was seen in 1 case (1.2%); type F (A4b) was present in 1 case (1.2%). When the composition of LSA contained PÍ of the double branch, the supplying range of the PÍ was further divided into type I (A4, A5) that was found in 1 patient (1.2%) and type II (A4a, A5) seen in 7 patients (8.5%).

**Table 7:** The supplying range of the PÍ.

	Our study (*n* = 82)	
	NO.	%
One branch		
A4 + A5	14	17.1
A5	44	53.7
A4a + A5	10	12.2
A5b	4	4.9
A4a	1	1.2
A4b	1	1.2
Two branches		
A4, A5	1	1.2
A4a, A5	7	8.5

**Figure 5:**
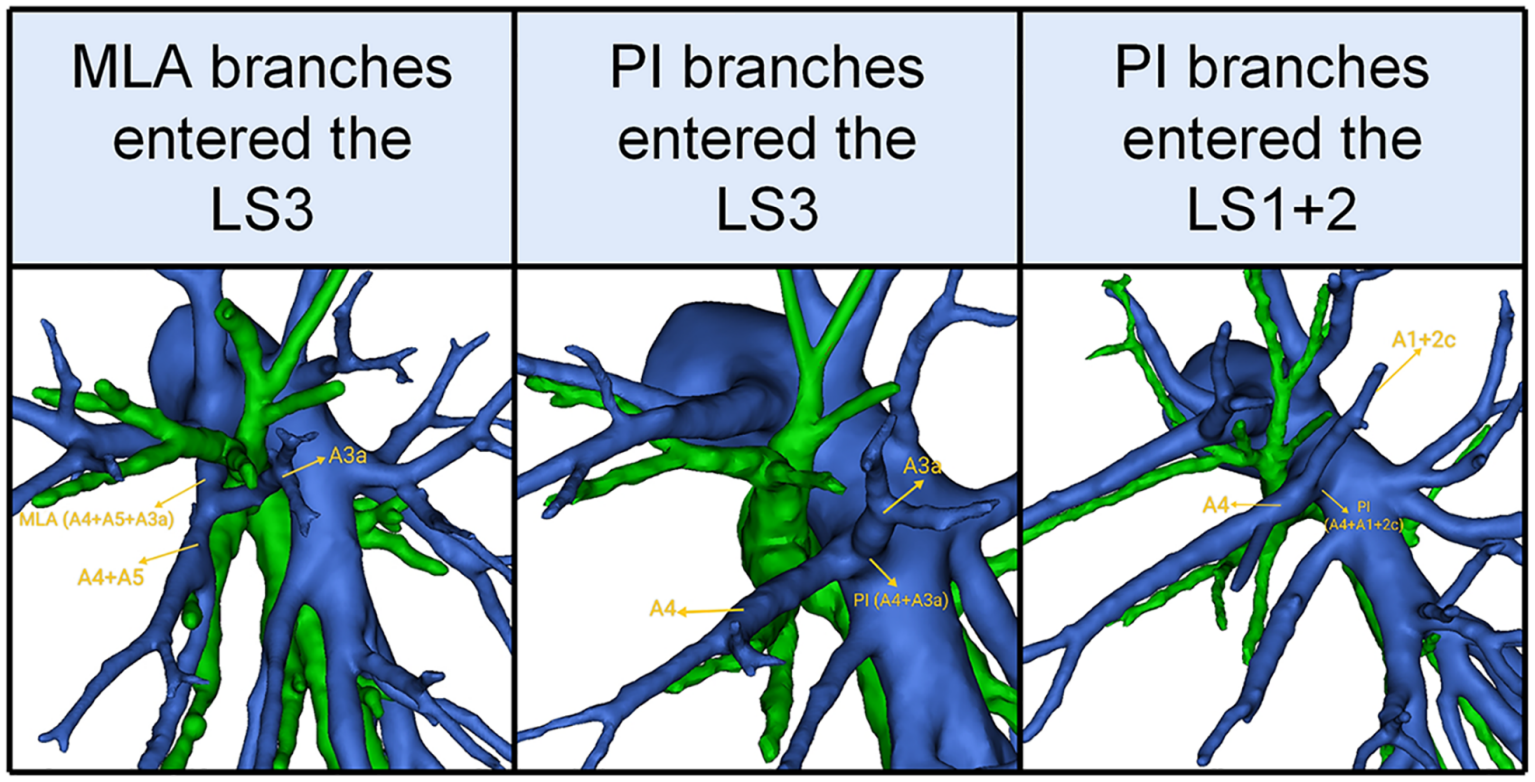
3D reconstruction model of the branches of the lingular segment artery entered the LS1 + 2 and LS3.

In the present study, the total incidence rate of the branching of LSA associated with the LS1 + 2 and LS3 (143/540, 26.4%) contained the following three types (Table 8, Figure 5): type I, the MLA branches entered the LS3a in 25 patients (25/540, 4.6%); type II, the PI branches entered the LS3a in 44 patients (44/540, 8.1%); type III, the PI branches entered the LS1 + 2c (74/540, 13.7%).

**Table 8:** The branches of the lingular segment artery entered the LS1 + 2 and LS3.

	Our study (*n* = 540)		Maki (*n* = 539)		Murota (*n* = 320)	
	NO.	%	NO.	%	NO.	%
MLA branches entered the LS3a	25	4.6	NR	-	NR	-
PI branches entered the LS3a	44	8.1	33	6.1	26	8.1
PI branches entered the LS1 + 2c	74	13.7	NR	-	12	3.8

NR, the type was not referred.

### Combinations of branching patterns of LSA and LSDV

As detailed in Table 9, there were 51 cases (9.4%) with Non-central vein type, 50 cases (9.3%) with Central vein type, 268 cases (49.6%) with Semi-central vein type in the interlobar type, and 7 cases (1.3%) with Non-central vein type, 9 cases (1.7%) with Central vein type, 29 cases (5.4%) with Semi-central vein type in the mediastinal type. Moreover, the Non-central vein type, the Central vein type, and the Semi-central vein type accounted for 18 (3.3%), 60 (11.1%), and 48 (8.9%) in the interlobar and mediastinal type. Combinations of the branching patterns of LSA and LSDV were significantly dependent (*p* < 0.005). This indicated that the combinations of the interlobar and mediastinal type with Central vein type, while the interlobar type and the mediastinal type with the Semi-central vein type were frequently observed (Table 9).

**Table 9:** Distribution of branching patterns in the left superior division veins between different lingular segment artery types.

	Non-central vein type	Central vein type	Semi-central vein type	Total	*p* value
Interlobar type	51	50	268	369	*p* < 0.005
Mediastinal type	7	9	29	45	
Interlobar and mediastinal type	18	60	48	126	
Total	76	119	345	540	

## Discussion

As the low-dose thin-slice chest CT is commonly applied for healthy examinations, the detection rate of GGOs is greatly improved. The JCOG0804 study, which estimated the efficacy and safety of sublobar resection for GGO-dominant peripheral lung cancer with a maximum tumor diameter ≤2 cm and consolidation tumor ratio ≤0.25, concluded that sublobar resection should be the preferred surgical procedure (1). Additionally, VATS anatomical segmentectomy preserves more lung function and minimizes lung volume loss (1–5).

Anatomical variations in the branching pattern of the peripheral arterial are the major challenge for thoracic surgeons. Each arterial branch must be accurately discriminated prior to ligature—especially when performing VATS anatomic segmentectomy. VATS anatomic segmentectomy demands a more comprehensive understanding of local anatomy than lobectomy. Knowledge of the LSA branching pattern is indispensable because the most common intraoperative catastrophe during VATS anatomic segmentectomy is an injury to the pulmonary artery (6, 7, 18). More recently, Louie and Cerfolio also illuminated the strategy of pulmonary arterial bleeding during robotic pulmonary resections and discovered the upper lobes, particularly the LUL, to be a common source of hemorrhage (19, 20).

Recent technical progress in medical imaging (especially 3D-CTBA) has promoted the preoperative identification of pulmonary bronchovascular patterns. Fukuhara estimated the efficacy of preoperative assessment of the pulmonary artery branches using 3D-CTBA and found 95.2% (139 of 146) of pulmonary artery branches were precisely identified intraoperatively (21). However, there are only a few reports that have comprehensively summarized and classified the bronchovascular pattern of LS using 3D-CTBA. In this study, we analyzed the bronchovascular patterns of the LS and emphasized the differences between our results and those of previous reports (8–16).

In this study, the origins of LSB were divided into two types (Table 1, Figure 1). B4 from the central division of the upper lobe bronchus and B5 from the inferior division of the upper lobe bronchus was a rare anomaly (4.4%), which has not been reported in the literature (8, 9, 12). Thus, it is extremely easy to mistake the inferior division of the upper lobe bronchus (B5) for B4 + B5 during LS segmentectomy, which will narrow the intersegmental plane. We found horizontally bifurcated lingular segment bronchus in 12 cases (2.2%), which was higher than that reported by Maki (1.5%) and Zhang (0.6%) (8, 9). This distinctive branching pattern is shown in Figure 1. In these cases, the location of B4 could finally be recognized by referring to the bifurcation pattern of LSA and LLDV.

The classification of the inflow destination of LLDV was the same as that of Maki (9) (Table 2, Figure 2A). However, it is significant to understand the inflow destination of LLDV for safe lobectomy. In the LUL lobectomy, if LLDV drains into LA, we should apply appropriate traction to the LUL in order to avoid damage to LLDV. In the left lower lobe (LLL) lobectomy, if the LLDV is damaged while it drains into LLPV, infarction and necrosis of the LUL might occur (22). Lymph node dissection at the interlobar is a standard surgical method for primary lung cancer in the LUL and LLL. Thus, we should pay attention to the LLDV draining into LLPV during LLL lobectomy and lymph node dissection at the interlobar. In a word, paying attention to the inflow destination of LLDV is important for all left lung lobectomies.

We found that the branching patterns of LSDV also had the following three types (Table 3, Figure 2B): Semi-central vein type (63.9%), Non-central vein type (14.1%), and Central vein type (22.0%). Chen reported 62.9%, 8.9%, and 28.2%, respectively (13). Our study is similar to previous studies on the branching patterns of LSDV. In the Non-central vein type, V1 + 2c and V1 + 2d share a common trunk and drains into LUPV below B3. For the LS segmentectomy, it was necessary to avoid causing damage to the common trunk of V1 + 2c and V1 + 2d when the MLA was dissected from the side of the hilum of the mediastinum. When the LS segmentectomy is performed for patients with the central vein type, unexpected vein injury or inaccurate ligation of drainage veins might result if the thoracic surgeon ignores the branching patterns of LSDV, which may lead to uncontrolled bleeding or severe lung edema that can be life-threatening (22, 23). Knowing the branching patterns of LSDV and precise identification of the preoperative 3D-CTBA is useful for all thoracic surgeons in order to perform a safe and precise LS segmentectomy.

The mediastinal type was observed in 8.3% of cases in our study (Table 4, Figure 3B), which was considerably higher than the frequency reported by Deng (12) (6.8%), Maki (9) (6.3%), and Chen (13) (5.7%). The incidence of the interlobar and mediastinal type is 23.3%, which is similar to that of Deng (12) (16.5%), Maki (9) (23.6%), and Chen (13) (20.8%). The interlobar type was occured in 369 cases (68.3%) and was the most common type. In their studies, Deng, Maki and Chen reported 76.7%, 70.1% and 73.5% incidence, respectively (9, 12, 13). However, the interlobar type and the interlobar and mediastinal type have not been further classified in the previous papers (9, 12, 13). In this paper, the interlobar type and the interlobar and mediastinal type were subdivided into seven subtypes and four subtypes, respectively (Table 4, Figures 3A,B).

**Figure 6:**
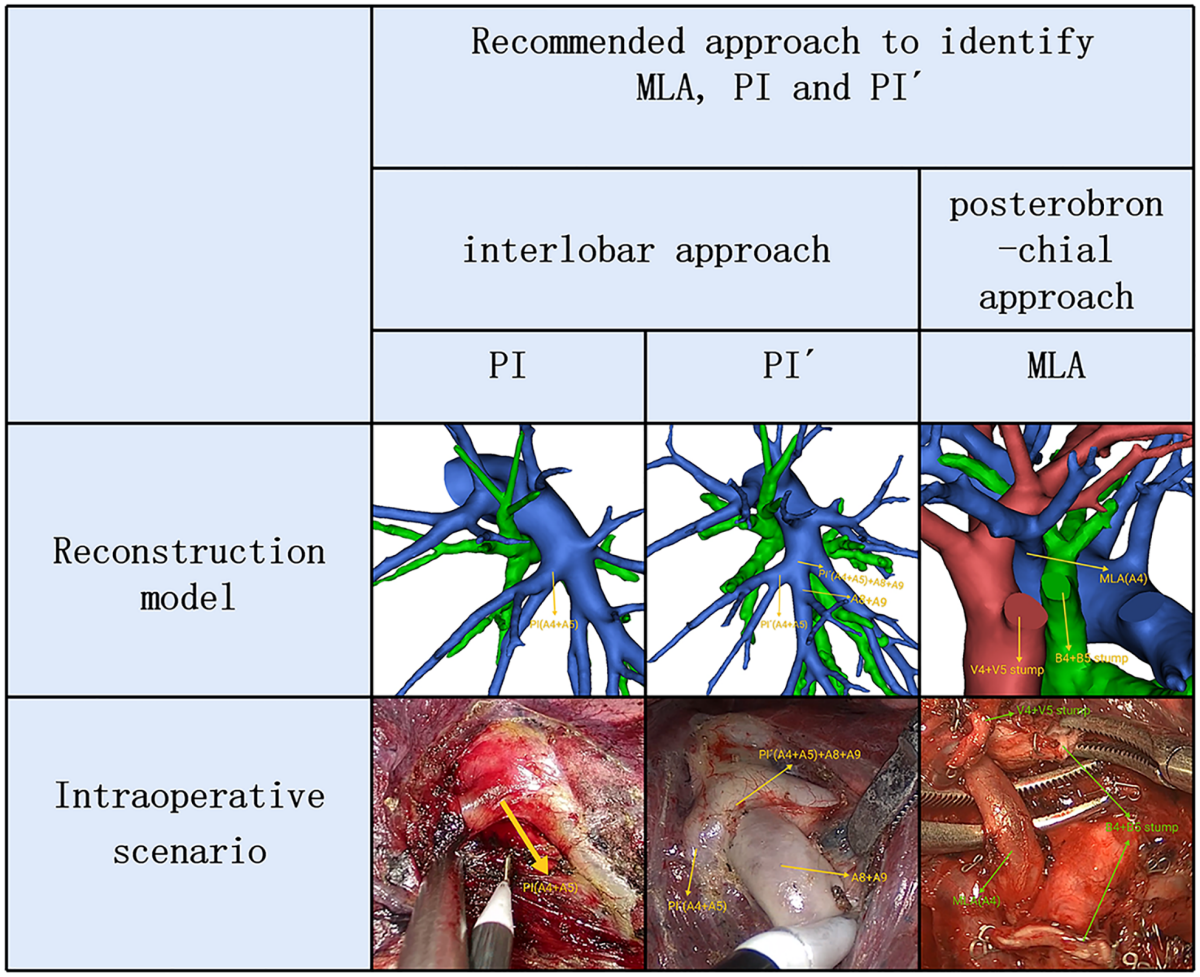
3D reconstruction and intraoperative scenario of two basic approaches identifying MLA, PI and PÍ.

Therefore, two basic approaches identifying MLA, PI and PÍ were first reported (Figure 6). The interlobar approach was used to identify artery branch that originate from the interlobar portion of the left pulmonary artery. The artery branch running deep within the LS was dissected using a posterobronchial approach. PI and PÍ can usually be discriminated by dissecting interlobar fissures (interlobar approach) (Figure 6). MLA was identified after resection of the LLDV and LSB from the side of the hilum of the mediastinum (posterobronchial approach) (Figure 6). The comprehensive understanding of two basic approaches and the branching pattern of LSA has clinical significance for accurate LS segmentectomy. If LSA was composed of PI and PÍ, PI and PÍ were dissected by adopting the interlobar approach. If LSA had two branches of MLA and PI, the posterobronchial approach and interlobar approach were respectively adopted to dissect MLA and PI.

In the present study, we found A4 + A5 was the most common type in the supplying range of the MLA (26.3%) (Table 5, Figure 4A). However, Wang reported that A4b was the most common type (11). The supplying range of the PI and PÍ were classified and summarized in detail (Tables 6, 7, Figures 4B–D). These classifications have not been reported previously. In the accurate LS segmentectomy, it is vital to determine how many branches there are and where each branch originates from. For example, for an accurate S4 segmentectomy, the intersegmental plane is easily altered without the knowledge of the supply range of MLA, PI, and PÍ. If MLA supplies S4 + S5a, a mistaken cut of it will result in an enlarged intersegmental plane. Similarly, if PI supplies S4 + S5, the artery branch supplying S5 should not be sacrificed. Moreover, the PÍ was seen in 82 cases in our study (15.2%). This compares with the figures presented by Wang (11) 24.1%, Murota (15) 29.7% and Maki (9) 5.8%, respectively. The preoperative identification of PÍ is significant. When LLL lobectomy is implemented, PÍ should not be ligated. In LUL lobectomy, if strong traction is applied to the LUL without recognizing the PÍ, we may damage the A8 + A9.

In addition, the branches of the LSA entered the LS1 + 2 and LS3 was seen in 26.4% of the cases (Table 8, Figure 5). A3a originating from PI accounted for 8.1% of cases, which was significantly higher than that reported by Maki (6.1%) (9). A1 + 2c came from PI in 13.7% of cases, which was significantly higher than that reported by Murota 3.8% and Wang 3.06% (11, 15). Moreover, we found A3a arose from MLA in 25 cases (4.6%), which has not been reported in the previous literature.

In the case of LS segmentectomy, it is necessary to observe whether A1 + 2c or A3a directly came from PI and MLA. When LS segmentectomy is performed in the case of A3a originating from MLA (Figure 5), A3a should not be resected.

There was a significant correlation between the branching patterns of LSA and LSDV (Table 9). No previous report has reported that the distribution of LSDV branching patterns between the mediastinal type and the interlobar and mediastinal type has a significant differences. Recently, Onuki have speculated a possibility of the axis rotation of LUL during the embryogenic period (24). In other words, With the growth of bronchi in the thorax, if the bronchus axis slightly rotates anticlockwise, the forward branch becomes B4, resulting in the mediastinal type. If the bronchus axis slightly rotates clockwise, the forward branch becomes B3, resulting in the interlobar type (24). Moreover, we also certified that the mediastinal type was often combined with the Semi-central vein type, and the interlobar and mediastinal type frequently appeared in the Central vein type. This implies that the mediastinal type and the interlobar and mediastinal type may result from different embryonic development.

## Conclusion

We analyzed the bronchovascular patterns of the LS in a much larger sample size (*n* = 540) than in the previously published reports. To our knowledge, this is the first report investigating the distribution of LSDV branching patterns between the mediastinal type and the interlobar and mediastinal type. We believe that the findings of the present study can be useful for thoracic surgeons when anatomical LS segmentectomy is planned and performed.

## Data Availability

The raw data supporting the conclusions of this article will be made available by the authors, without undue reservation.
